# Supplementary material for: A Ta-TaS2 monolith catalyst with robust and metallic interface for superior hydrogen evolution
Source: Nat Commun. 2021 Oct 18;12:6051. doi: 10.1038/s41467-021-26315-7 (PMC8523547; doi:10.1038/s41467-021-26315-7)
Supplement: Supplementary file 1 — Supplementary Information [file 41467_2021_26315_MOESM1_ESM.pdf]

Supplementary Information

A Ta-TaS_2 monolith catalyst with robust and metallic interface for superior hydrogen evolution

Qiangmin Yu[1], Zhiyuan Zhang[1], Siyao Qiu[2], Yuting Luo[1], Zhibo Liu[3], Fengning Yang[1], Heming Liu[1], Shiyu Ge[1], Xiaolong Zou[1], Baofu Ding[1], Wencai Ren[3], Hui-Ming Cheng[1, 3, 4], Chenghua Sun[2, 5*], and Bilu Liu[1*]

1. Shenzhen Geim Graphene Center, Tsinghua-Berkeley Shenzhen Institute & Institute of Materials Research, Tsinghua Shenzhen International Graduate School, Tsinghua University, Shenzhen 518055, P. R. China
2. College of Chemical Engineering and Energy Technology, Dongguan University of Technology, Dongguan 523808, P. R. China
3. Shenyang National Laboratory for Materials Sciences, Institute of Metal Research, Chinese Academy of Sciences, Shenyang, Liaoning, 110016, P. R. China
4. Advanced Technology Institute, University of Surrey, Guildford, Surrey GU27XH, UK
5. Department of Chemistry and Biotechnology, and Center for Translational Atomaterials, Swinburne University of Technology, Hawthorn, Victoria 3122, Australia

Corresponding authors

Email: bilu.liu@sz.tsinghua.edu.cn (B. L.)

chenghuasun@swin.edu.au (C. S.)

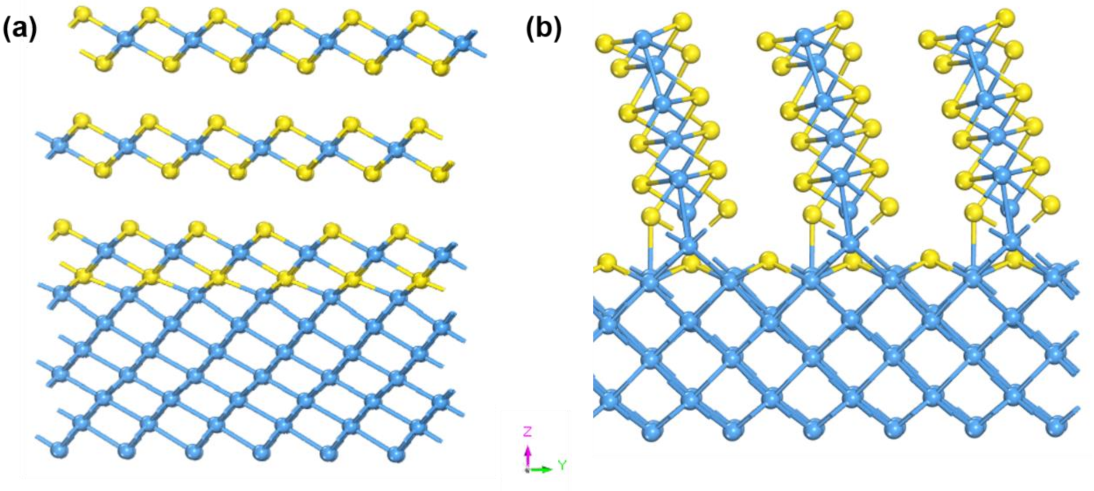

Supplementary Figure 1. (a) The atomic model of the MC with a parallel layer structure (denoted as Ta/TaS_2). (b) The atomic model of the MC with a perpendicular structure (Ta-TaS_2). In (a), the 2D TMDC nanosheets lie parallel to the metal substrate with only the bottom layer strongly anchored to the substrate, so that electrons need to get over the van der Waals gap between adjacent layers to reach active sites, resulting in additional resistance.

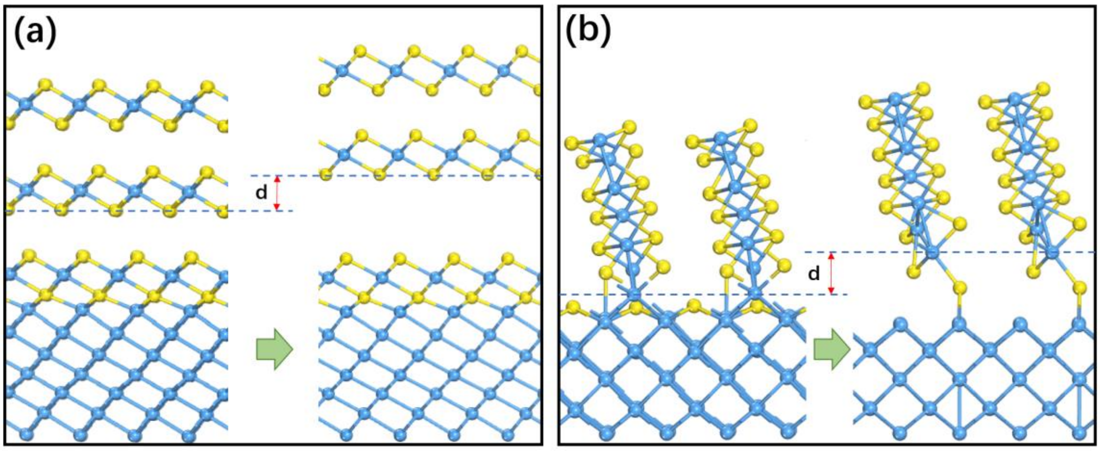

Supplementary Figure 2. The model for the mechanical strength calculations. (a) Ta/TaS_2 with van der Waals interaction; (b) Ta-TaS_2 MC with covalent bonds. To understand the mechanical strength, the energy evolution *E* with the distance *d* between Ta and TaS_2 has been investigated based on parallel (Fig. S2a) and vertical (Fig. S2b) stacking models, labelling as Ta/TaS_2 and Ta-TaS_2 MC in the main text. For Ta/TaS_2, interfacial separation needs to overcome van der Waals interaction, which is small due to the non-bonding nature. In the case of Ta-TaS_2 MC, as a contrast, the separation needs to break Ta-S covalent bonds, whose strength is significantly larger than the van der Waals interaction presented in Ta/TaS_2 interface. With respect to equilibrium distance as a reference, an energy cost of 0.37 eV and 10.56 eV is resulted to achieve full separation of Ta/TaS_2 and Ta-TaS_2 MC, respectively, as demonstrated in Fig.1d in the main text.

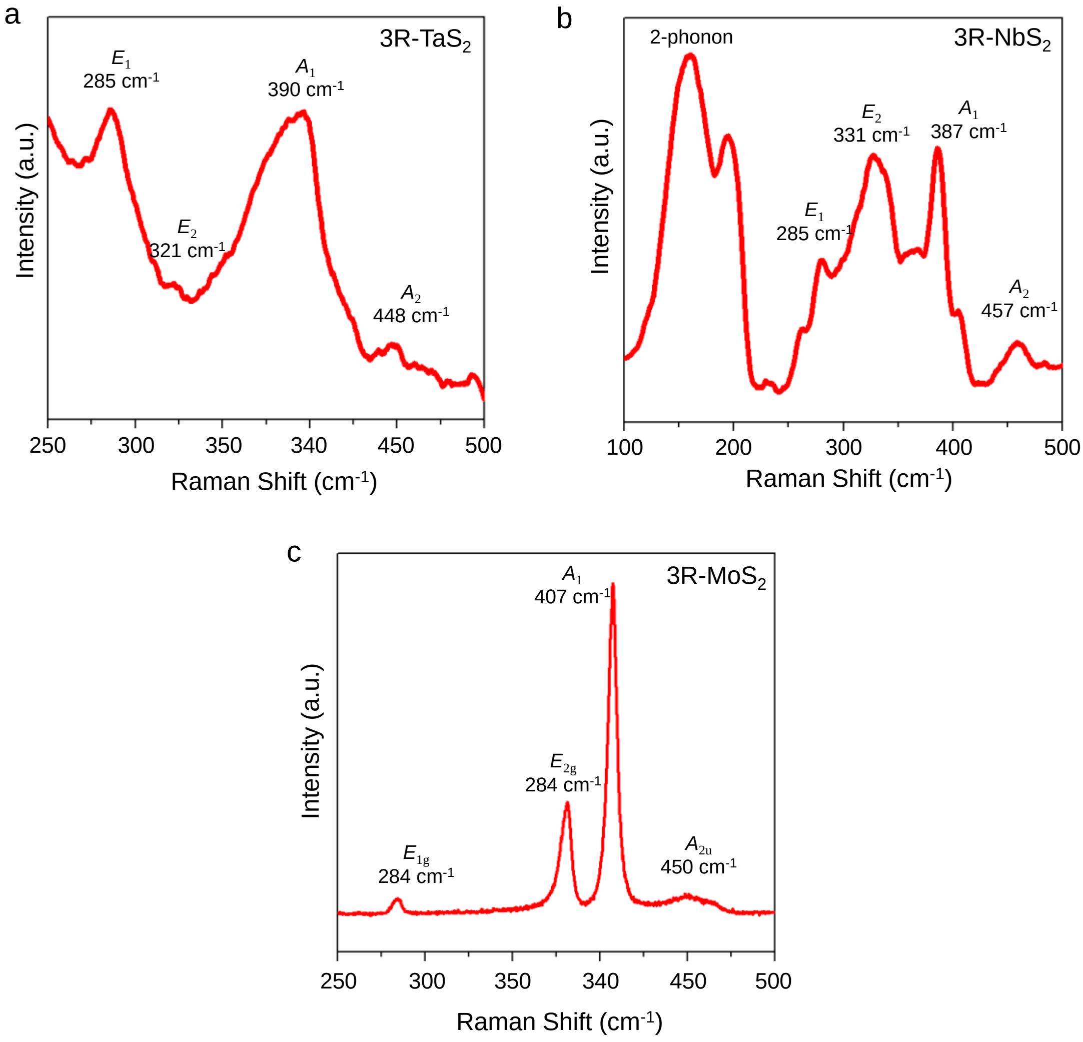

Supplementary Figure 3. Raman spectra of (a) 3R-TaS_2, (b) 3R-NbS_2, and (c) 3R-MoS_2.

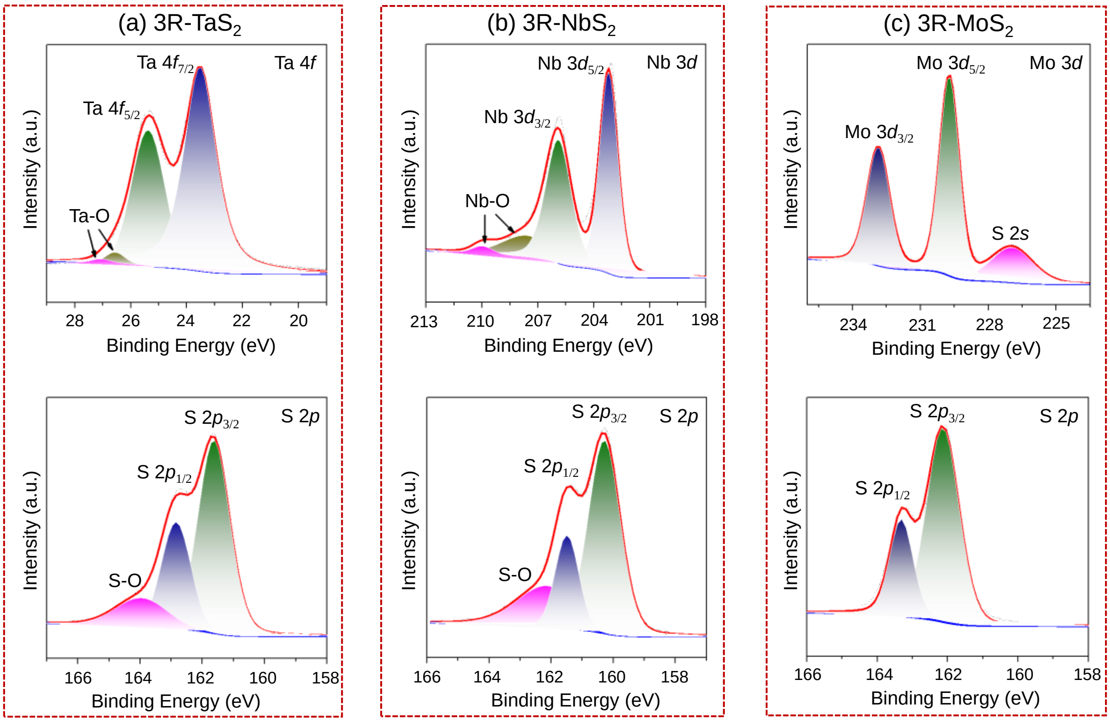

Supplementary Figure 4. XPS spectra of (a) 3R-TaS_2, (b) 3R-NbS_2, and (c) 3R-MoS_2.

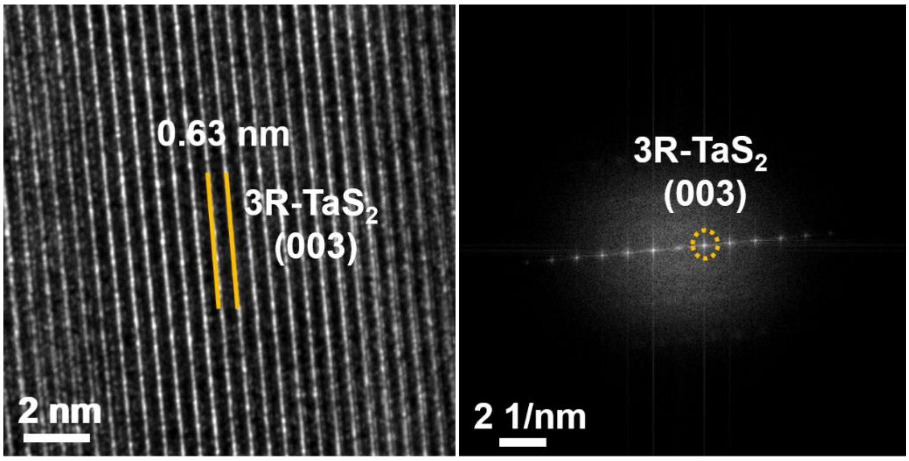

Supplementary Figure 5. Cross-sectional HRTEM image of 3R-TaS_2 and corresponding SEAD pattern.

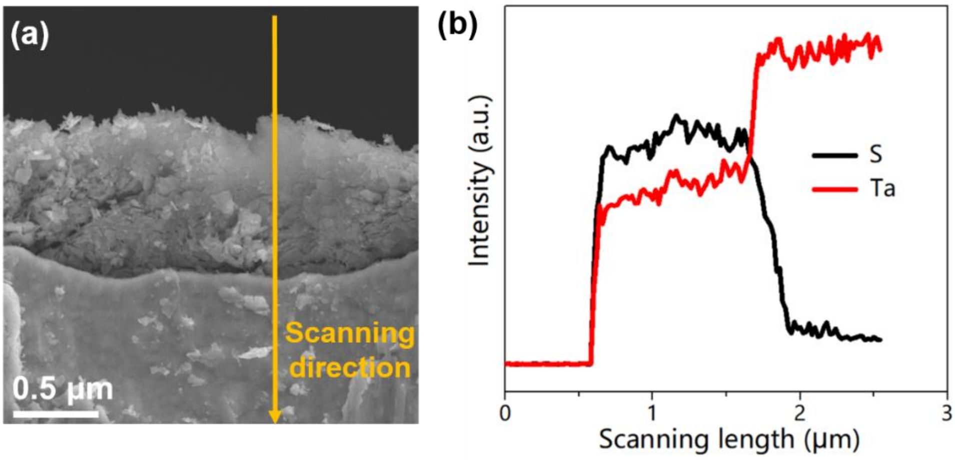

Supplementary Figure 6. (a) Cross-sectional SEM image and (b) the corresponding scans of the S and Ta elemental compositions of Ta-TaS_2 MC.

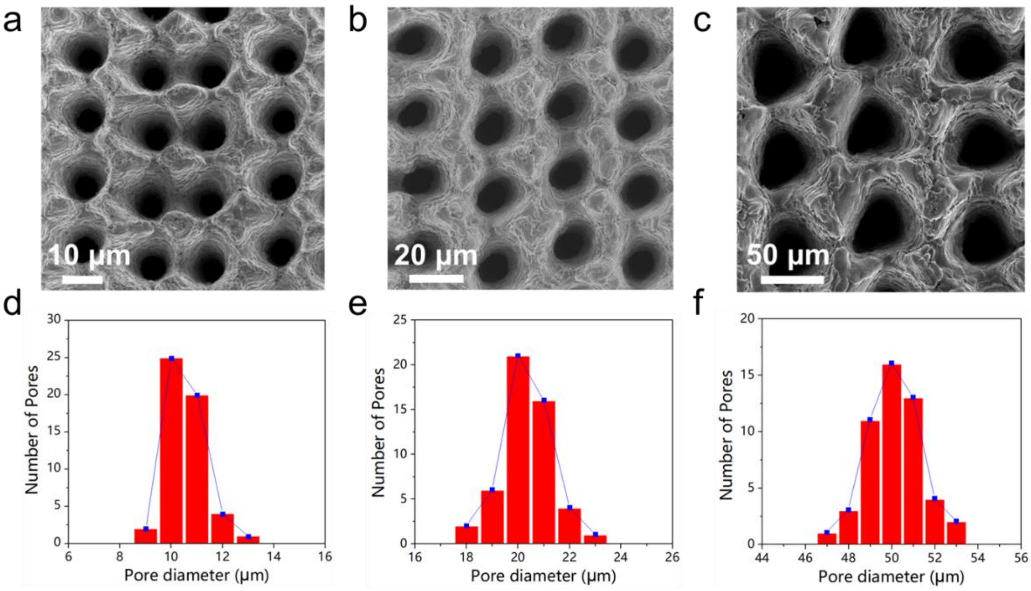

Supplementary Figure 7. (a-c) Different pore sizes on the Ta foil and (d-f) corresponding pore diameter distribution.

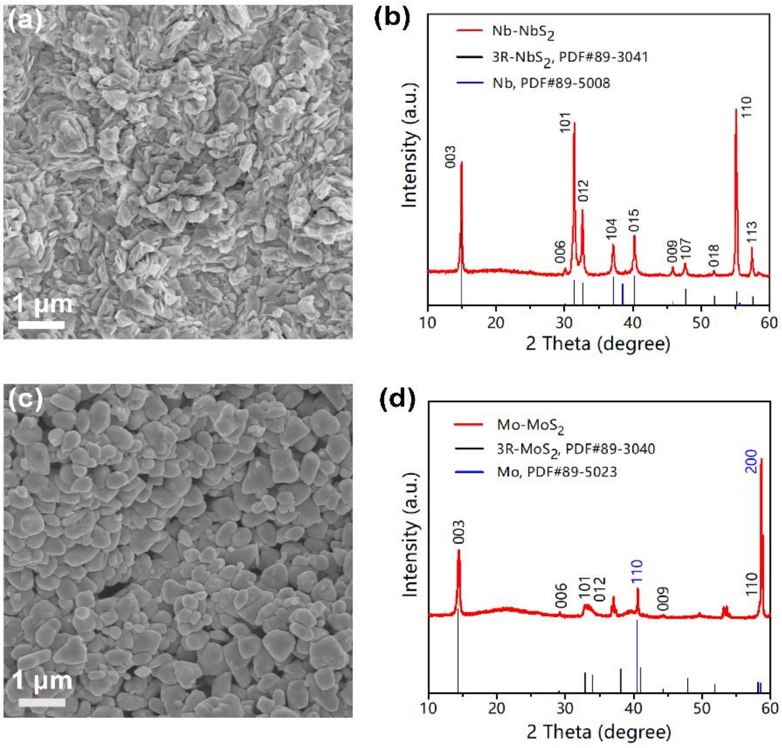

Supplementary Figure 8. SEM images and XRD patterns of (a-b) the Nb-NbS_2 MC and (c-d) the Mo-MoS_2 MC.

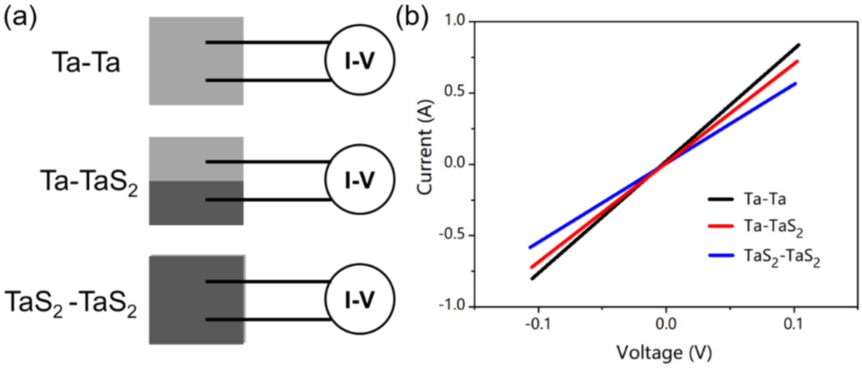

Supplementary Figure 9. (a) Diagram of the conductivity tests and (b) the corresponding I-V curves of the Ta foil, Ta-TaS_2 MC and TaS_2 materials. A conductivity of ~ 3×10^6 S/m was obtained for the Ta-TaS_2 material, which is comparable to the values for Ta metal (4×10^6 S/m) and metallic TaS_2 (2.8×10^6 S/m) material. Therefore, it is a near-zero resistance interface between Ta substrate and TaS_2 in Ta-TaS_2 monolith catalyst.

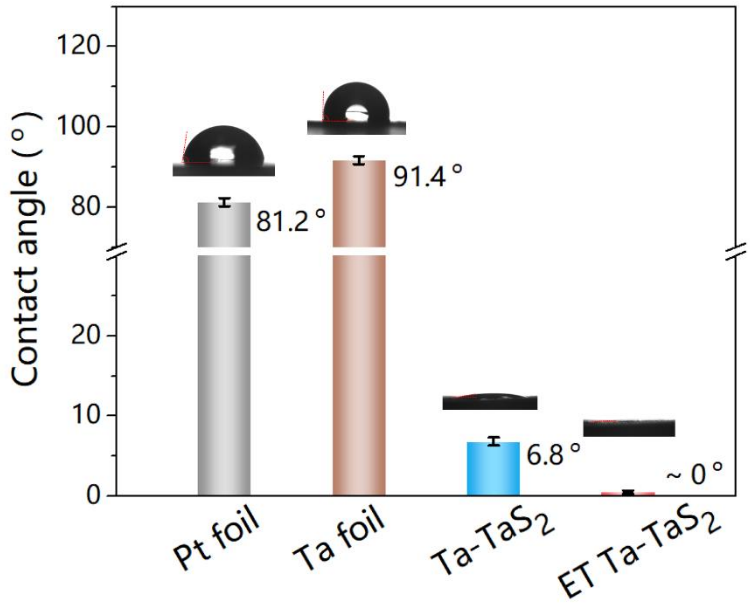

Supplementary Figure 10. Contact angles of a H_2O droplet on the surfaces of different samples, including a Pt foil, a Ta foil, Ta-TaS_2 MC, and an electrochemically treated (ET) Ta-TaS_2 MC. The contact angles were measured for at least three times for each sample, and error bars correspond to standard deviations. The error bars show the standard derivation.

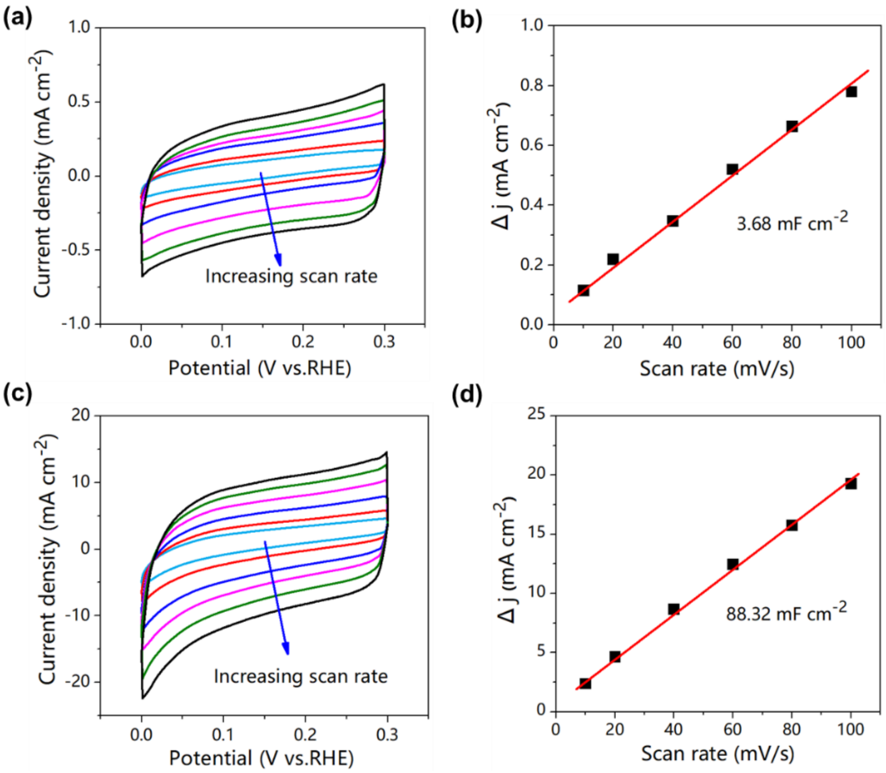

Supplementary Figure 11. CV curves obtained with different scan rates from 10 to 100 mV s^{-1} in a 0.5 M H_2SO_4 electrolyte and a capacitive current of 0.15 V against the scan rate and corresponding C_{dl} values estimated by linear fitting of the plots for Ta-TaS_2 MC. (a-b) after 500 CV cycles; (c-d) after 15,000 CV cycles.

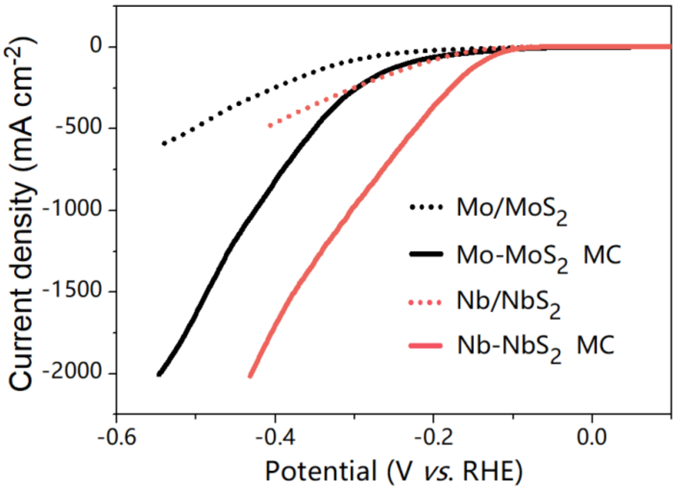

Supplementary Figure 12. Polarization curves of different samples including Nb-NbS_2 MC, Nb/NbS_2 composite, Mo-MoS_2 MC, and Mo/MoS_2 composite measured in a 0.5 M H_2SO_4 electrolyte. The electrochemical results showed that the Nb-NbS_2 MC and Mo-MoS_2 MC achieved a current density of 1,750 mA cm^{-2} and 826 mA cm^{-2} at the overpotential of 400 mV, which is much larger than that of the Nb/NbS_2 composite (463 mA cm^{-2}) and Mo/MoS_2 composite (242 mA cm^{-2}) at the same overpotential. The excellent performance of these MCs synthesized by the OSPS method stems from the excellent charge transfer kinetic in MC and robust interface between substrate and catalysts. These results support the importance of the OSPS method in synthesizing high performance Nb-NbS_2 and Mo-MoS_2 based monolith catalysts.

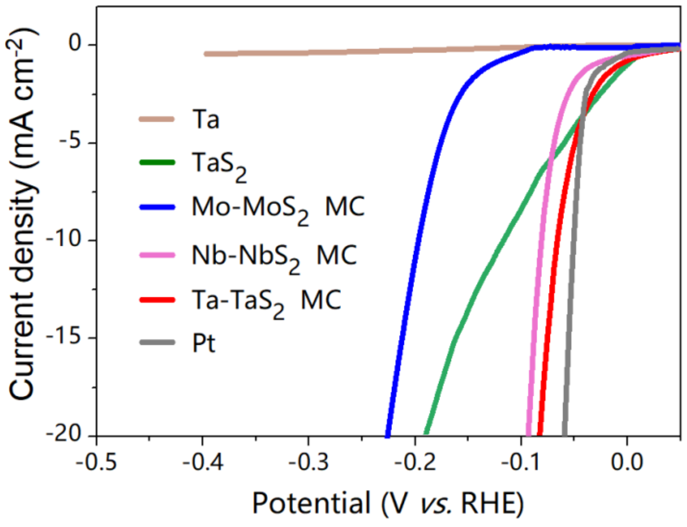

Supplementary Figure 13. Polarization curves of different samples under small current densities.

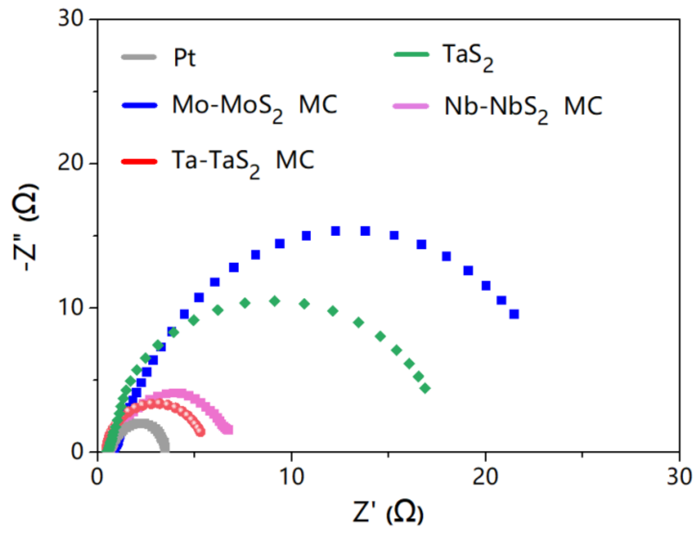

Supplementary Figure 14. Electrochemical impedance measurements of different samples at an overpotential of 50 mV. The results showed a solution resistance of ~ 0.2 Ω and a charge transfer resistance of ~ 3.2 Ω, suggesting excellent charge transfer in Ta-TaS_2 monolith catalyst.

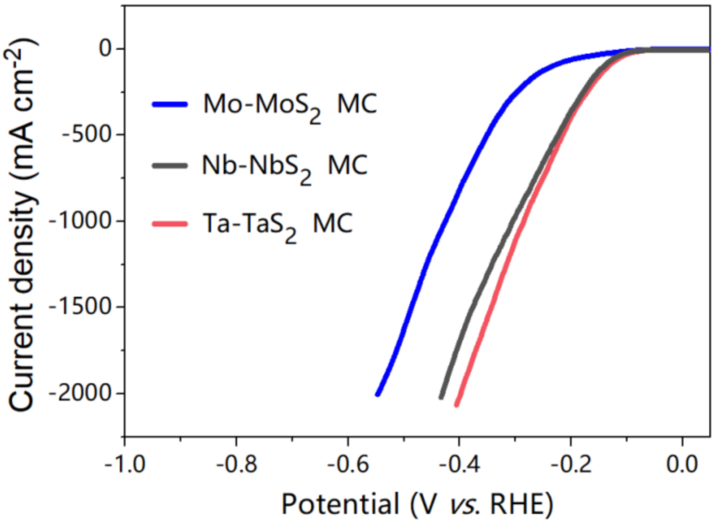

Supplementary Figure 15. Polarization curves of Ta-TaS_2 MC, Nb-NbS_2 MC and Mo-MoS_2 MC measured in a 0.5 M H_2SO_4 electrolyte.

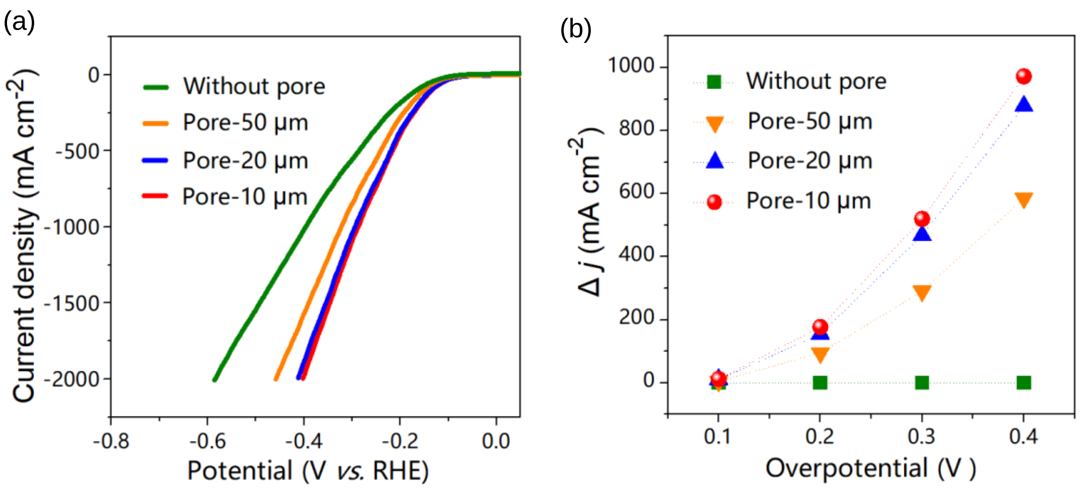

Supplementary Figure 16. (a) Polarization curves of Ta-TaS_2 MC samples with different pore sizes and (b) comparison of their corresponding activities. Note: Δj represents the current density of a porous MC sample minus the current density of a MC sample without pore.

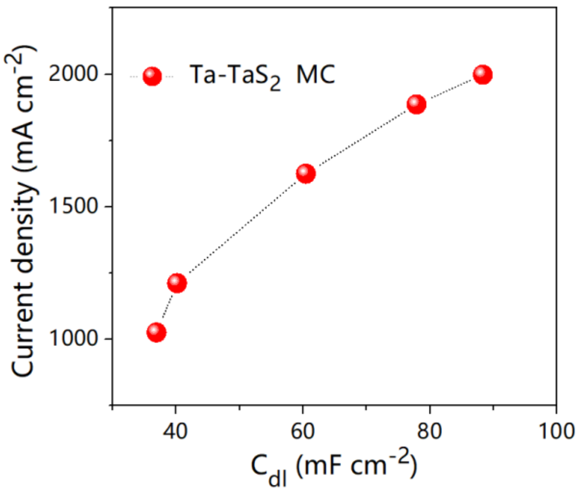

Supplementary Figure 17. Catalytic performance *vs.* capacitance of double-layer (C_{dl}) of the Ta-TaS_2 MC sample at -0.4 V.

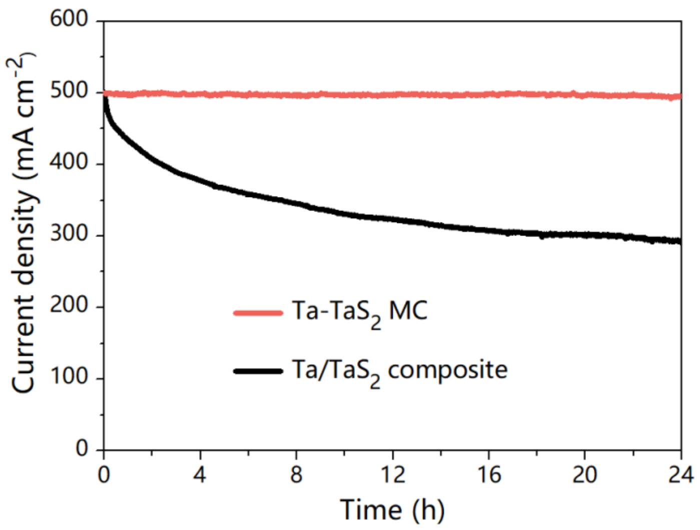

Supplementary Figure 18. I-T curves of Ta/TaS$_2$ composite catalyst and Ta-TaS$_2$ MC. The stability of Ta/TaS$_2$ composite catalyst shows a significant decrease after 24 h test at an overpotential of 355 mV, which is much worse than that of Ta-TaS$_2$ monolith catalyst, indicating a robust interface built between Ta and TaS$_2$ in Ta-TaS$_2$ MC.

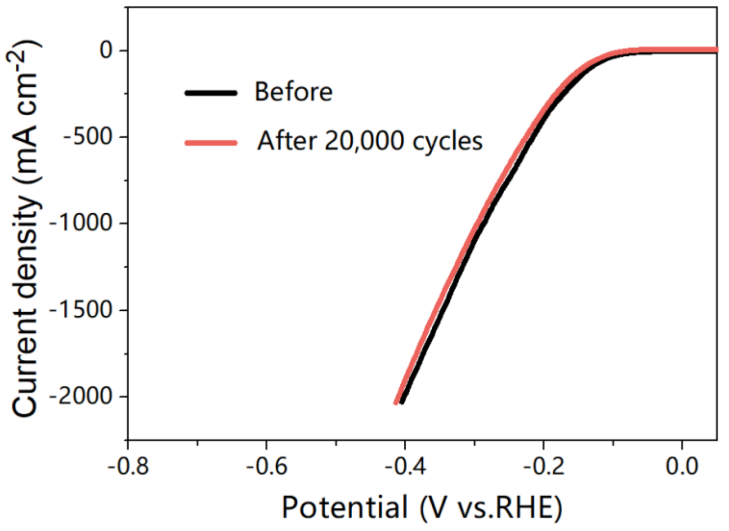

Supplementary Figure 19. Polarization curves of the as-synthesized Ta-TaS$_2$ MC catalyst and after 20,000 cycles.

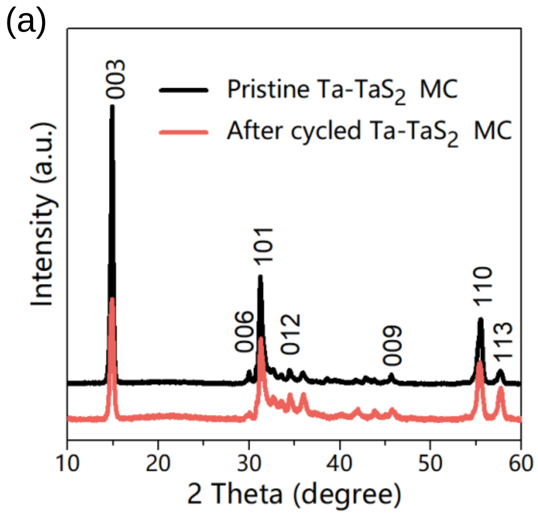

Supplementary Figure 20. (a) XRD patterns and (b) XPS spectra of the Ta-TaS_2 MC before and after 20,000 cycles test.

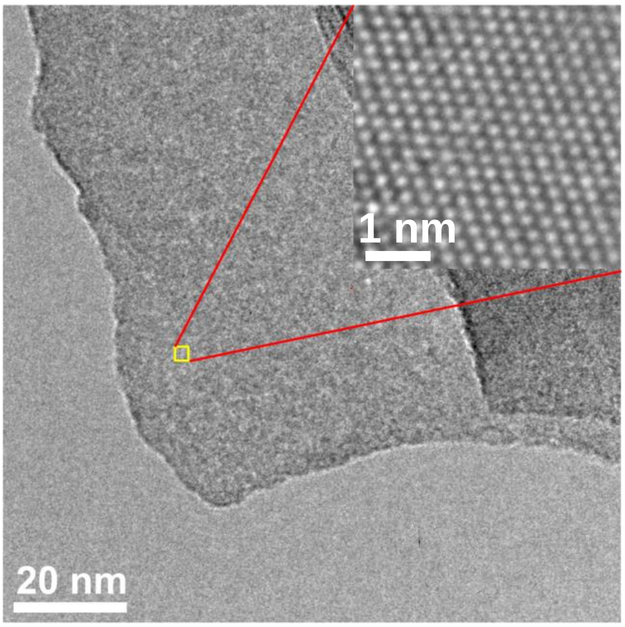

Supplementary Figure 21. TEM and HRTEM (inset) images of a Ta-TaS_2 MC after 20,000 cycles.

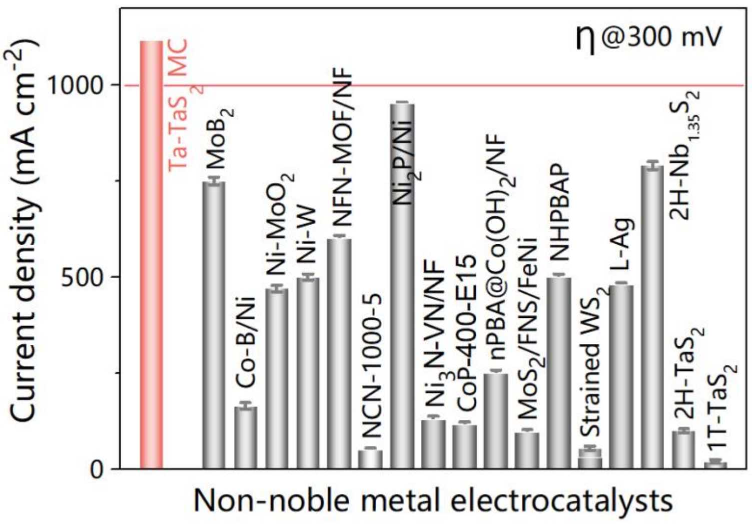

Supplementary Figure 22. HER performance comparisons with the state-of-the-art reported literatures from different kinds of non-noble metal catalysts at an overpotential of 300 mV.

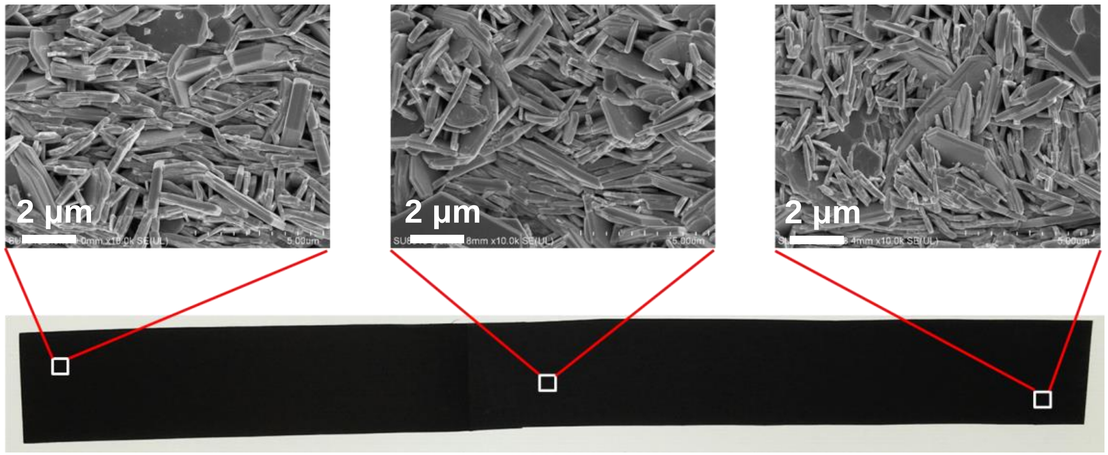

Supplementary Figure 23. Optical image of a large-area Ta-TaS_2 MC and corresponding SEM images captured at different regions, revealing the uniform growth of TaS_2 on the Ta foil.

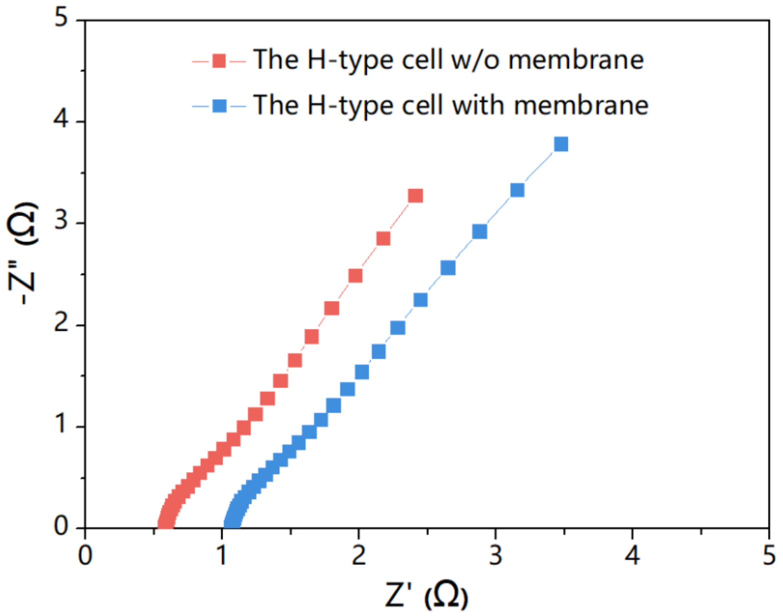

Supplementary Figure 24. Electrochemical impedance measurements of the H-type cells with and w/o membrane at a constant potential of 1.55 V. The solution resistance is 0.6 Ω and the membrane resistance is < 0.5 Ω in this work.

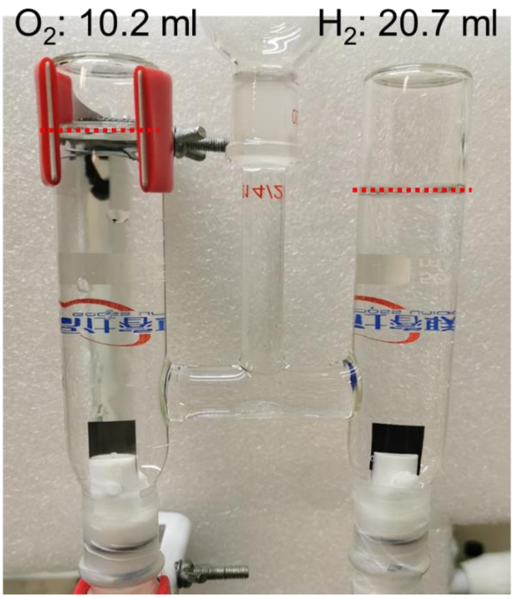

Supplementary Figure 25. Optical image of the home-made electrochemical cell used for hydrogen collection and evaluation of the Faraday efficiency.

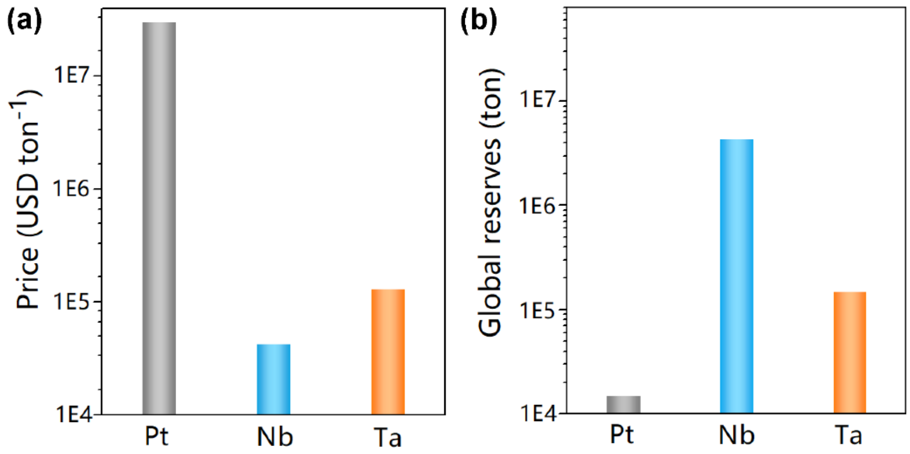

Supplementary Figure 26. (a)The price and (b) global reserves of Pt, Nb, and Ta metals.

Supplementary Table 1. The electrical conductance of different materials.

Materials	Electrical conductance (S/m)	Reference
**Iridium (Ir)**	1.9×10^7	Ref. S1
**Platinum (Pt)**	9.4×10^6	Ref. S2
**TaS_2**	3.3×10^6	Ref. S3
**Graphite**	1.3×10^5	Ref. S4
**MoS_2**	12	Ref. S5
**NiO**	1×10^{-3}	Ref. S6
**Ta foil**	4.0×10^6	Our material
**3R-TaS_2**	2.8×10^6	Our material
**Ta-TaS_2 MC**	3.5×10^6	Our material

Supplementary Table 2. A comparison of the HER performance of TaS_2 materials.

Samples (Phase)	η@10 mA cm^{-2} (mV)	Tafel slopes (mV dec^{-1})	Stability	References
**TaS_2 (2H)**	65-150	45-49	N/A	Ref. R7
**Au/TaS_2 (2H)**	101	53	12 h @10 mA cm^{-2}	Ref. R8
**Ta-TaS_2 (3R)**	65	30	200 h @200-1000 mA cm^{-2}	This work

Supplementary Table 3. Comprehensive comparisons of the HER performance of Ta-TaS_2 MC obtained in this work with those reported in literature.

Catalysts	The largest test current (mA)	Current density(mA cm^{-2}) (η=300 mV)	The stability test current density (mA cm^{-2})	The stability test time (h)	Refs
**Co/Se-MoS_2-NF**	1000	~ 630	1000	360	Ref. S9
**NFN-MOF/NF**	600	~ 595	100	30	Ref. S12
**Ni_3N-VN/NF**	200	~ 120	10	20	Ref. S14
**CoP-400-E15**	500	~ 100	500	90	Ref. S15
**MoS_2/FNS/FeNi**	150	~ 150	10	10	Ref. S16
**NHPBAP**	500	~ 500	100	20	Ref. S17
**Strained WS_2**	100	~ 55	30	120	Ref. S18
**L-Ag**	500	~ 480	10	160	Ref. S19
**2H-$Nb_{1.35}S_2$**	1000	~ 800	500	130	Ref. S21
**Ni NP|Ni–N–C/E**	50	~32	30	10	Ref. S22
**Co-B/Ni**	500	~165	50	20	Ref. S10
**Ni-W**	700	~ 500	10	30	Ref. S11
**NCN-1000-5**	50	~ 50	20	~4	Ref. S13
**2H-TaS_2**	160	~ 100	N/A	N/A	Ref. S8
**2H-NbS_2**	200	N/A	N/A	N/A	Ref. S3
**1T-TaS_2**	120	~ 22	30	24	Ref. S20
**Nb-NbS_2 MC**	2000	995	N/A	N/A	This work
**Ta-TaS_2 MC**	2000	1115	200-1000	200	This work

Supplementary References

1. Bell, Terence. Electrical conductivity of metals. thoughtco.com/electrical-conductivity-in-metals-2340117 (2020).
2. Liu K, Kang X, Zhou Z-Y, Song Y, Lee LJ, Tian D*, et al.* Platinum nanoparticles functionalized with acetylene derivatives: electronic conductivity and electrocatalytic activity in oxygen reduction. *J. Electroanalyt. Chem.*, **688,** 143-150 (2013).
3. Liu Y, Wu J, Hackenberg KP, Zhang J, Wang YM, Yang Y*, et al.* Self-optimizing, highly surface-active layered metal dichalcogenide catalysts for hydrogen evolution. *Nat. Energ.*, **2**, 17127 (2017).
4. Ji H, Zhang L, Pettes MT, Li H, Chen S, Shi L*, et al.* Ultrathin graphite foam: a three-dimensional conductive network for battery electrodes. *Nano Lett.*, **12**, 2446-2451 (2012).
5. Gopalakrishnan K, Sultan S, Govindaraj A, Rao CNR. Supercapacitors based on composites of PANI with nanosheets of nitrogen-doped RGO, $BC_{1.5}N$, MoS_2 and WS_2. *Nano Energ.*, **12**, 52-58 (2015).
6. Pramanik P, and Bhattacharya S. A chemical method for the deposition of nickel oxide thin films. *J. Electrochem. Soc.*, **137** 3869 (1990).
7. Shi J, Wang X, Zhang S, Xiao L, Huan Y, Gong Y*, et al.* Two-dimensional metallic tantalum disulfide as a hydrogen evolution catalyst. *Nat. Commun.*, **8**, 958 (2017).
8. Yu Q, Luo Y, Qiu S, Li Q, Cai Z, Zhang z, *et al.* Tuning the hydrogen evolution performance of metallic 2D tantalum disulfide by interfacial engineering. *ACS Nano*, **13**, 11874-11881 (2019).
9. Zheng Z, Yu L, Gao M, Chen X, Zhou W, Ma C*, et al.* Boosting hydrogen evolution on MoS_2 via co-confining selenium in surface and cobalt in inner layer. *Nat. Commun.*, **11**, 3315 (2020).
10. Hao W, Wu R, Zhang R, Ha Y, Chen Z, Wang L*, et al.* Electroless plating of highly efficient bifunctional boride-based electrodes toward practical overall water splitting. *Adv. Energ. Mater.*, **8**, 1801372 (2018).
11. Wu H, Kong L, Ji Y, Yan J, Ding Y, Li Y*, et al.* Double-site Ni–W nanosheet for best alkaline HER performance at high current density > 500 mA cm^{-2}. *Adv. Mater. Interf.*, **6**, 1900308 (2019).
12. Senthil Raja D, Chuah X-F, Lu S-Y. In situ grown bimetallic MOF-based composite as highly efficient bifunctional electrocatalyst for overall water splitting with ultrastability at high current densities. *Adv. Energ. Mater.*, **8**, 1801065 (2018).
13. Jiang H, Gu J, Zheng X, Liu M, Qiu X, Wang L*, et al.* Defect-rich and ultrathin N doped carbon nanosheets as advanced trifunctional metal-free electrocatalysts for the ORR, OER and HER. *Energ. Environ. Sci.*, **12**, 322-333 (2019).
14. Yan H, Xie Y, Wu A, Cai Z, Wang L, Tian C*, et al.* Anion-modulated HER and OER activities of 3D Ni–V-based interstitial compound heterojunctions for high-efficiency and stable overall water splitting. *Adv. Mater.*, **31**, 1901174 (2019).
15. Yu X, Wang M, Gong X, Guo Z, Wang Z, Jiao S. Self-supporting porous CoP-based films with phase-separation structure for ultrastable overall water electrolysis at large current density. *Adv. Energ. Mater.*, **8**, 1802445 (2018).
16. Wu Y, Li F, Chen W, Xiang Q, Ma Y, Zhu H*, et al.* Coupling interface constructions of $MoS_2/Fe_5Ni_4S_8$ heterostructures for efficient electrochemical water splitting. *Adv. Mater.*, **30**,

1803151 (2018).

17. Ge Y, Dong P, Craig SR, Ajayan PM, Ye M, Shen J. Transforming nickel hydroxide into 3D prussian blue analogue array to obtain Ni_2P/Fe_2P for efficient hydrogen evolution reaction. *Adv. Energ. Mater.*, **8**, 1800484 (2018).

18. Voiry, D., Yamaguchi, H., Li, J. *et al*. Enhanced catalytic activity in strained chemically exfoliated WS_2 nanosheets for hydrogen evolution. *Nat. Mater.*, **12**, 850–855 (2013).

19. Li Z, Fu J-Y, Feng Y, Dong C-K, Liu H, Du X-W. A silver catalyst activated by stacking faults for the hydrogen evolution reaction. *Nat. Catal.*, **2**, 1107-1114 (2019).

20. Huan Y, Shi J, Zou X, Gong Y, Zhang Z, Li M*, et al.* Vertical 1T-TaS_2 synthesis on nanoporous gold for high-performance electrocatalytic applications. *Adv. Mater.*, **30**, 1705916 (2018).

21. Yang J, Mohmad AR, Wang Y, Fullon R, Song X, Zhao F, *et al.* Ultrahigh-current-density niobium disulfide catalysts for hydrogen evolution. *Nat. Mater.*, **18**, 1309–1314 (2019).

22. Lei C, Wang Y, Hou Y, Liu P, Yang J, Zhang T, *et al.* Efficient alkaline hydrogen evolution on atomically dispersed Ni–N_x Species anchored porous carbon with embedded Ni nanoparticles by accelerating water dissociation kinetic. *Energ. Environ. Sci.*, **12**, 149 (2019).
